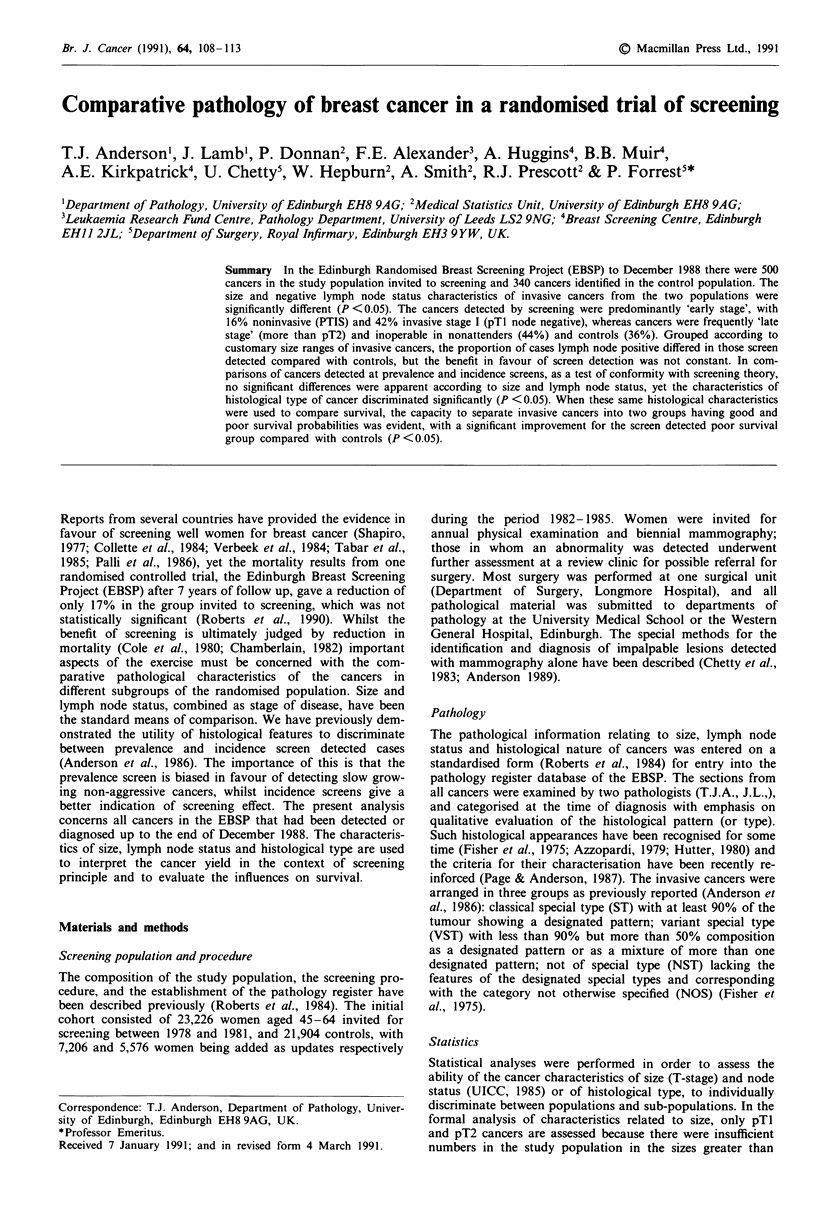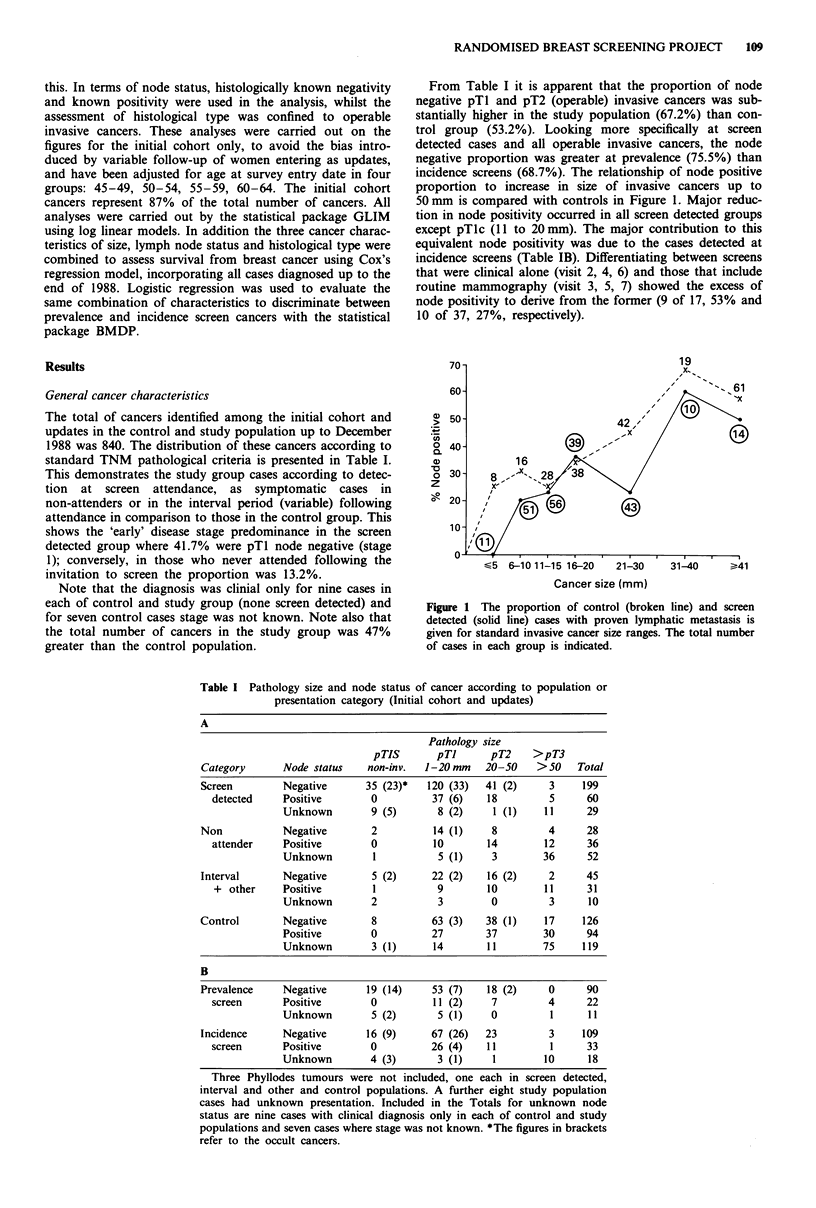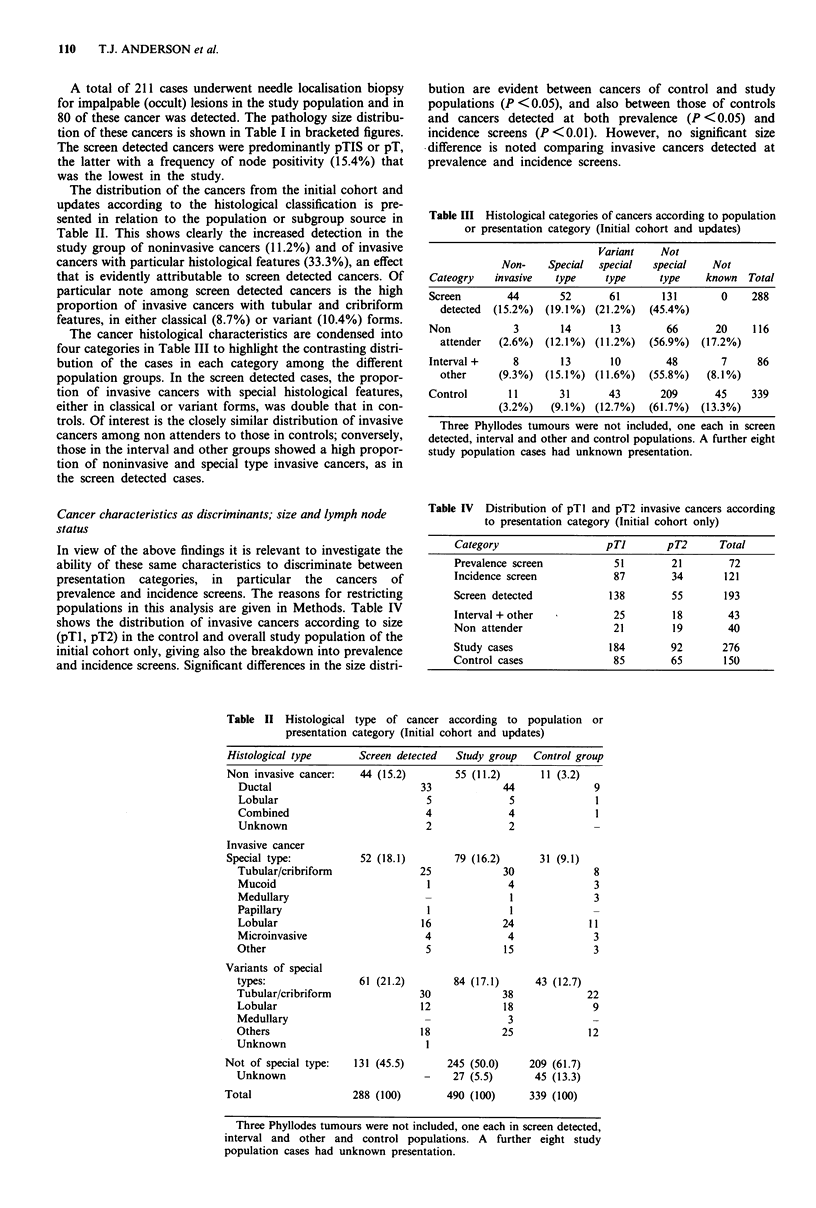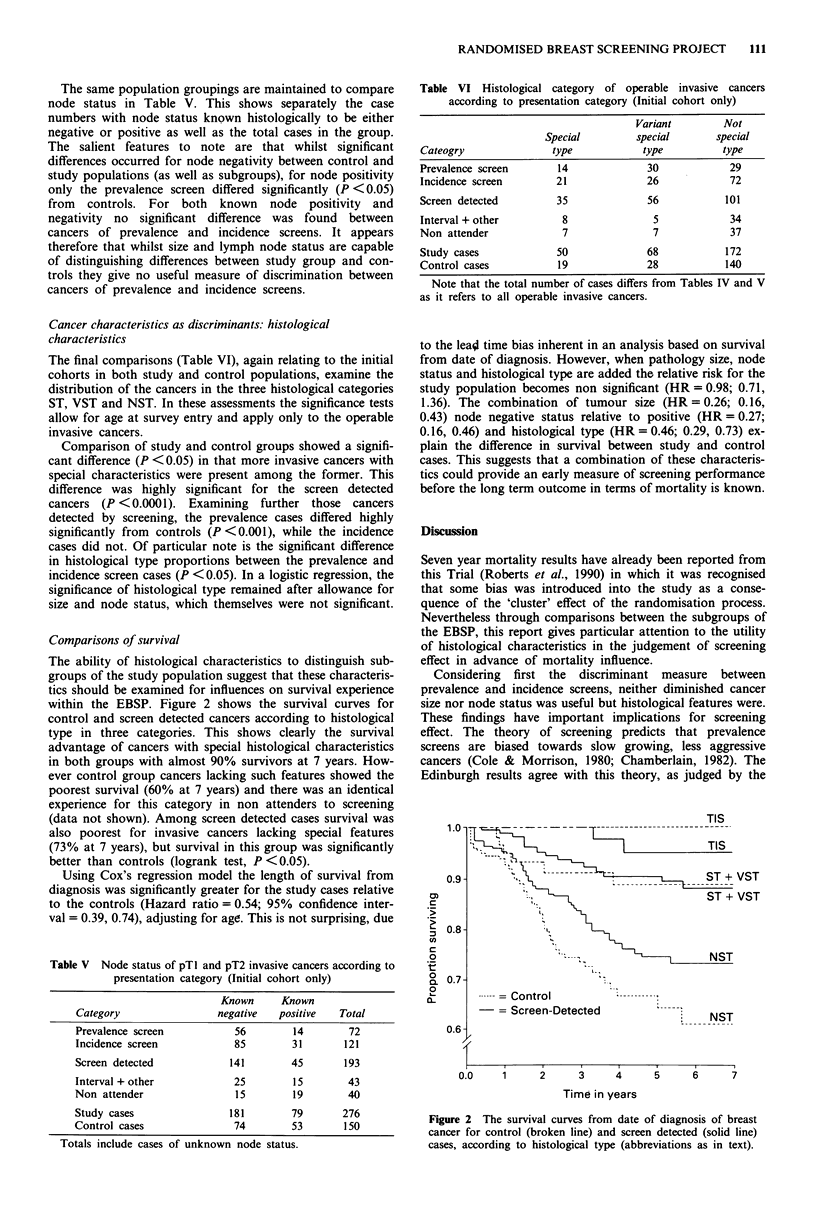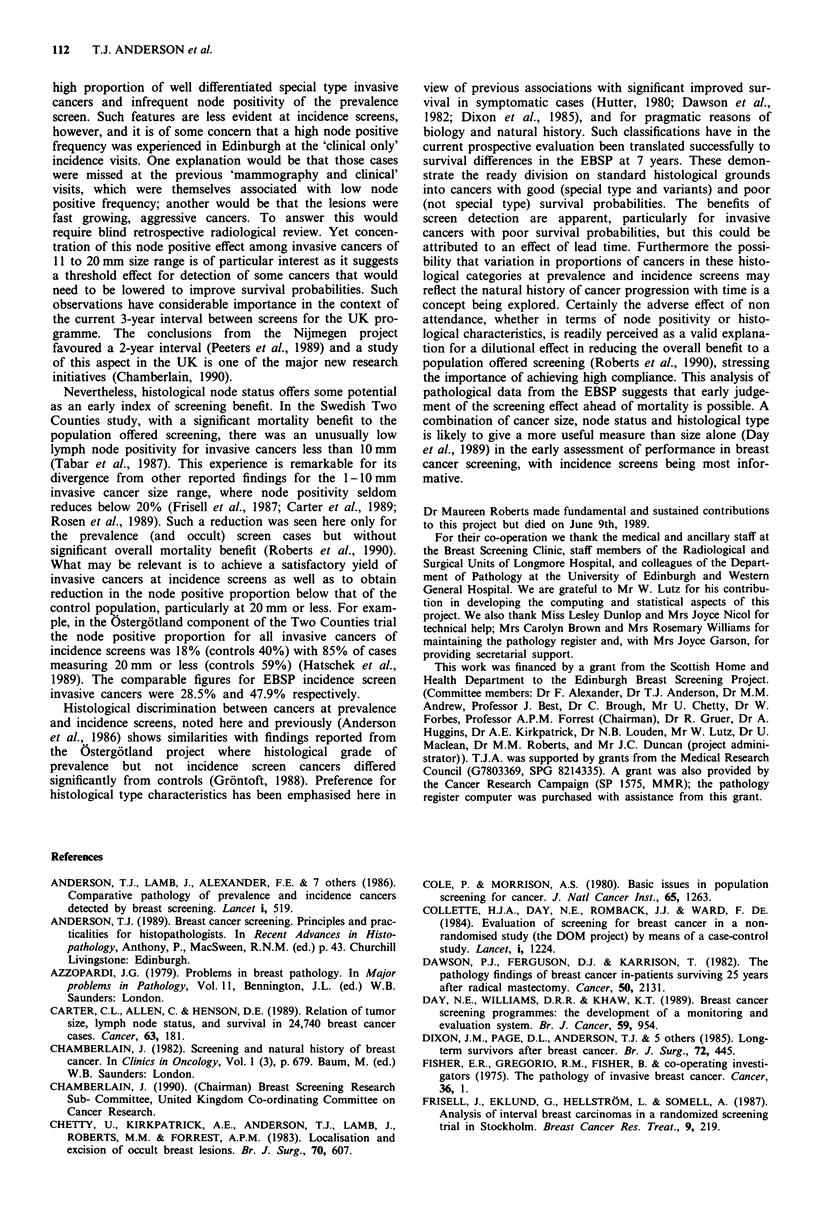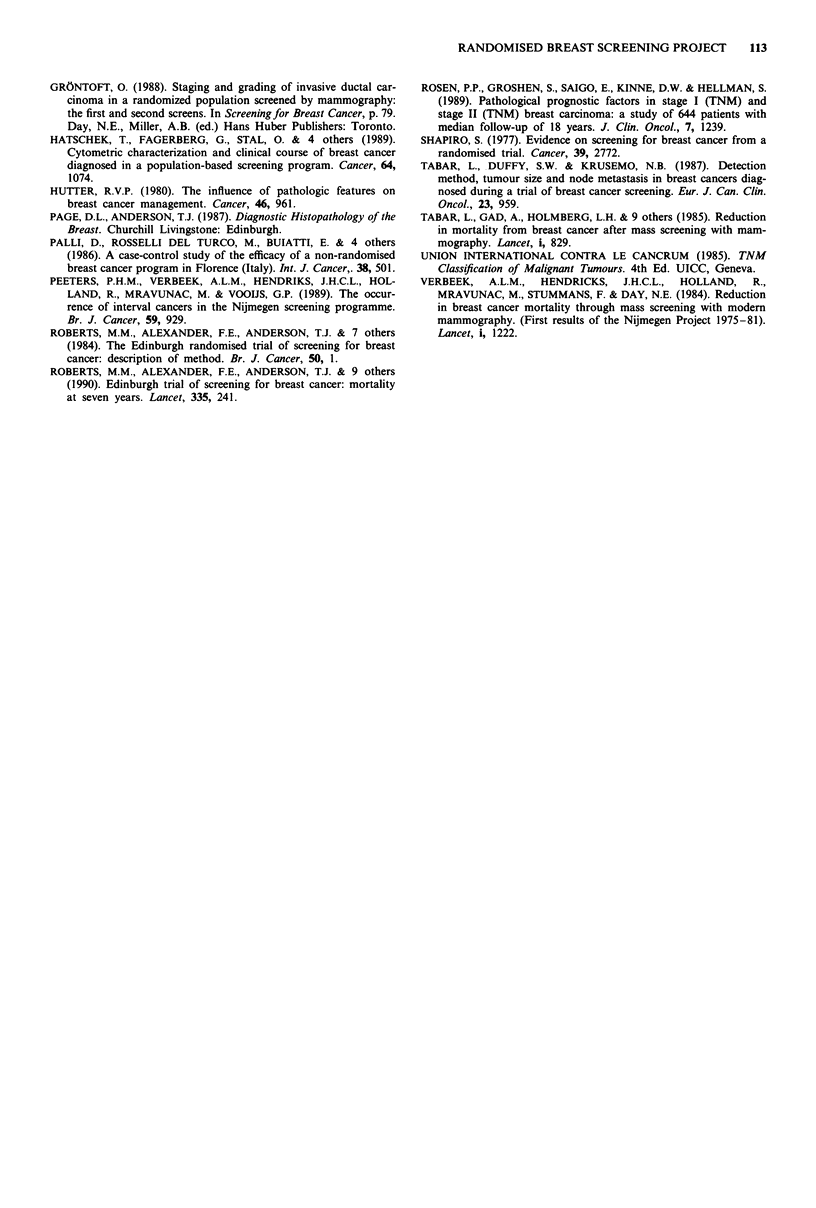# Comparative pathology of breast cancer in a randomised trial of screening.

**DOI:** 10.1038/bjc.1991.251

**Published:** 1991-07

**Authors:** T. J. Anderson, J. Lamb, P. Donnan, F. E. Alexander, A. Huggins, B. B. Muir, A. E. Kirkpatrick, U. Chetty, W. Hepburn, A. Smith

**Affiliations:** Department of Pathology, University of Edinburgh, UK.

## Abstract

In the Edinburgh Randomised Breast Screening Project (EBSP) to December 1988 there were 500 cancers in the study population invited to screening and 340 cancers identified in the control population. The size and negative lymph node status characteristics of invasive cancers from the two populations were significantly different (P less than 0.05). The cancers detected by screening were predominantly 'early stage', with 16% noninvasive (PTIS) and 42% invasive stage I (pT1 node negative), whereas cancers were frequently 'late stage' (more than pT2) and inoperable in nonattenders (44%) and controls (36%). Grouped according to customary size ranges of invasive cancers, the proportion of cases lymph node positive differed in those screen detected compared with controls, but the benefit in favour of screen detection was not constant. In comparisons of cancers detected at prevalence and incidence screens, as a test of conformity with screening theory, no significant differences were apparent according to size and lymph node status, yet the characteristics of histological type of cancer discriminated significantly (P less than 0.05). When these same histological characteristics were used to compare survival, the capacity to separate invasive cancers into two groups having good and poor survival probabilities was evident, with a significant improvement for the screen detected poor survival group compared with controls (P less than 0.05).


Br. J. Cancer (1991), 64, 108–113 © Macmillan Press Ltd., 1991

### Comparative pathology of breast cancer in a randomised trial of screening

T.J. Anderson[1], J. Lamb[1], P. Donnan[2], F.E. Alexander[3], A. Huggins[4], B.B. Muir[4], A.E. Kirkpatrick[4], U. Chetty[5], W. Hepburn[2], A. Smith[2], R.J. Prescott[2] & P. Forrest[5]*

[1]*Department of Pathology, University of Edinburgh EH8 9AG;* [2]*Medical Statistics Unit, University of Edinburgh EH8 9AG;* [3]*Leukaemia Research Fund Centre, Pathology Department, University of Leeds LS2 9NG;* [4]*Breast Screening Centre, Edinburgh EH11 2JL;* [5]*Department of Surgery, Royal Infirmary, Edinburgh EH3 9YW, UK.*

**Summary** In the Edinburgh Randomised Breast Screening Project (EBSP) to December 1988 there were 500 cancers in the study population invited to screening and 340 cancers identified in the control population. The size and negative lymph node status characteristics of invasive cancers from the two populations were significantly different ($P < 0.05$). The cancers detected by screening were predominantly 'early stage', with 16% noninvasive (PTIS) and 42% invasive stage I (pT1 node negative), whereas cancers were frequently 'late stage' (more than pT2) and inoperable in nonattenders (44%) and controls (36%). Grouped according to customary size ranges of invasive cancers, the proportion of cases lymph node positive differed in those screen detected compared with controls, but the benefit in favour of screen detection was not constant. In comparisons of cancers detected at prevalence and incidence screens, as a test of conformity with screening theory, no significant differences were apparent according to size and lymph node status, yet the characteristics of histological type of cancer discriminated significantly ($P < 0.05$). When these same histological characteristics were used to compare survival, the capacity to separate invasive cancers into two groups having good and poor survival probabilities was evident, with a significant improvement for the screen detected poor survival group compared with controls ($P < 0.05$).

Reports from several countries have provided the evidence in favour of screening well women for breast cancer (Shapiro, 1977; Collette *et al.*, 1984; Verbeek *et al.*, 1984; Tabar *et al.*, 1985; Palli *et al.*, 1986), yet the mortality results from one randomised controlled trial, the Edinburgh Breast Screening Project (EBSP) after 7 years of follow up, gave a reduction of only 17% in the group invited to screening, which was not statistically significant (Roberts *et al.*, 1990). Whilst the benefit of screening is ultimately judged by reduction in mortality (Cole *et al.*, 1980; Chamberlain, 1982) important aspects of the exercise must be concerned with the comparative pathological characteristics of the cancers in different subgroups of the randomised population. Size and lymph node status, combined as stage of disease, have been the standard means of comparison. We have previously demonstrated the utility of histological features to discriminate between prevalence and incidence screen detected cases (Anderson *et al.*, 1986). The importance of this is that the prevalence screen is biased in favour of detecting slow growing non-aggressive cancers, whilst incidence screens give a better indication of screening effect. The present analysis concerns all cancers in the EBSP that had been detected or diagnosed up to the end of December 1988. The characteristics of size, lymph node status and histological type are used to interpret the cancer yield in the context of screening principle and to evaluate the influences on survival.

### Materials and methods

### *Screening population and procedure*

The composition of the study population, the screening procedure, and the establishment of the pathology register have been described previously (Roberts *et al.*, 1984). The initial cohort consisted of 23,226 women aged 45–64 invited for screening between 1978 and 1981, and 21,904 controls, with 7,206 and 5,576 women being added as updates respectively during the period 1982–1985. Women were invited for annual physical examination and biennial mammography; those in whom an abnormality was detected underwent further assessment at a review clinic for possible referral for surgery. Most surgery was performed at one surgical unit (Department of Surgery, Longmore Hospital), and all pathological material was submitted to departments of pathology at the University Medical School or the Western General Hospital, Edinburgh. The special methods for the identification and diagnosis of impalpable lesions detected with mammography alone have been described (Chetty *et al.*, 1983; Anderson 1989).

### *Pathology*

The pathological information relating to size, lymph node status and histological nature of cancers was entered on a standardised form (Roberts *et al.*, 1984) for entry into the pathology register database of the EBSP. The sections from all cancers were examined by two pathologists (T.J.A., J.L.,), and categorised at the time of diagnosis with emphasis on qualitative evaluation of the histological pattern (or type). Such histological appearances have been recognised for some time (Fisher *et al.*, 1975; Azzopardi, 1979; Hutter, 1980) and the criteria for their characterisation have been recently reinforced (Page & Anderson, 1987). The invasive cancers were arranged in three groups as previously reported (Anderson *et al.*, 1986): classical special type (ST) with at least 90% of the tumour showing a designated pattern; variant special type (VST) with less than 90% but more than 50% composition as a designated pattern or as a mixture of more than one designated pattern; not of special type (NST) lacking the features of the designated special types and corresponding with the category not otherwise specified (NOS) (Fisher *et al.*, 1975).

### *Statistics*

Statistical analyses were performed in order to assess the ability of the cancer characteristics of size (T-stage) and node status (UICC, 1985) or of histological type, to individually discriminate between populations and sub-populations. In the formal analysis of characteristics related to size, only pT1 and pT2 cancers are assessed because there were insufficient numbers in the study population in the sizes greater than

Correspondence: T.J. Anderson, Department of Pathology, University of Edinburgh, Edinburgh EH8 9AG, UK.
*Professor Emeritus.
Received 7 January 1991; and in revised form 4 March 1991.

this. In terms of node status, histologically known negativity and known positivity were used in the analysis, whilst the assessment of histological type was confined to operable invasive cancers. These analyses were carried out on the figures for the initial cohort only, to avoid the bias introduced by variable follow-up of women entering as updates, and have been adjusted for age at survey entry date in four groups: 45–49, 50–54, 55–59, 60–64. The initial cohort cancers represent 87% of the total number of cancers. All analyses were carried out by the statistical package GLIM using log linear models. In addition the three cancer characteristics of size, lymph node status and histological type were combined to assess survival from breast cancer using Cox's regression model, incorporating all cases diagnosed up to the end of 1988. Logistic regression was used to evaluate the same combination of characteristics to discriminate between prevalence and incidence screen cancers with the statistical package BMDP.

### Results

### *General cancer characteristics*

The total of cancers identified among the initial cohort and updates in the control and study population up to December 1988 was 840. The distribution of these cancers according to standard TNM pathological criteria is presented in Table I. This demonstrates the study group cases according to detection at screen attendance, as symptomatic cases in non-attenders or in the interval period (variable) following attendance in comparison to those in the control group. This shows the 'early' disease stage predominance in the screen detected group where 41.7% were pT1 node negative (stage 1); conversely, in those who never attended following the invitation to screen the proportion was 13.2%.

Note that the diagnosis was clinial only for nine cases in each of control and study group (none screen detected) and for seven control cases stage was not known. Note also that the total number of cancers in the study group was 47% greater than the control population.

From Table I it is apparent that the proportion of node negative pT1 and pT2 (operable) invasive cancers was substantially higher in the study population (67.2%) than control group (53.2%). Looking more specifically at screen detected cases and all operable invasive cancers, the node negative proportion was greater at prevalence (75.5%) than incidence screens (68.7%). The relationship of node positive proportion to increase in size of invasive cancers up to 50 mm is compared with controls in Figure 1. Major reduction in node positivity occurred in all screen detected groups except pT1c (11 to 20 mm). The major contribution to this equivalent node positivity was due to the cases detected at incidence screens (Table IB). Differentiating between screens that were clinical alone (visit 2, 4, 6) and those that include routine mammography (visit 3, 5, 7) showed the excess of node positivity to derive from the former (9 of 17, 53% and 10 of 37, 27%, respectively).

**Figure 1** The proportion of control (broken line) and screen detected (solid line) cases with proven lymphatic metastasis is given for standard invasive cancer size ranges. The total number of cases in each group is indicated.

**Table I** Pathology size and node status of cancer according to population or presentation category (Initial cohort and updates)

| Category | Node status | *pTIS non-inv.* | *Pathology size pT1 1–20 mm* | *pT2 20–50* | *>pT3 >50* | *Total* |
|---|---|---|---|---|---|---|
| **A** | | | | | | |
| Screen detected | Negative | 35 (23)* | 120 (33) | 41 (2) | 3 | 199 |
| | Positive | 0 | 37 (6) | 18 | 5 | 60 |
| | Unknown | 9 (5) | 8 (2) | 1 (1) | 11 | 29 |
| Non attender | Negative | 2 | 14 (1) | 8 | 4 | 28 |
| | Positive | 0 | 10 | 14 | 12 | 36 |
| | Unknown | 1 | 5 (1) | 3 | 36 | 52 |
| Interval + other | Negative | 5 (2) | 22 (2) | 16 (2) | 2 | 45 |
| | Positive | 1 | 9 | 10 | 11 | 31 |
| | Unknown | 2 | 3 | 0 | 3 | 10 |
| Control | Negative | 8 | 63 (3) | 38 (1) | 17 | 126 |
| | Positive | 0 | 27 | 37 | 30 | 94 |
| | Unknown | 3 (1) | 14 | 11 | 75 | 119 |
| **B** | | | | | | |
| Prevalence screen | Negative | 19 (14) | 53 (7) | 18 (2) | 0 | 90 |
| | Positive | 0 | 11 (2) | 7 | 4 | 22 |
| | Unknown | 5 (2) | 5 (1) | 0 | 1 | 11 |
| Incidence screen | Negative | 16 (9) | 67 (26) | 23 | 3 | 109 |
| | Positive | 0 | 26 (4) | 11 | 1 | 33 |
| | Unknown | 4 (3) | 3 (1) | 1 | 10 | 18 |

Three Phyllodes tumours were not included, one each in screen detected, interval and other and control populations. A further eight study population cases had unknown presentation. Included in the Totals for unknown node status are nine cases with clinical diagnosis only in each of control and study populations and seven cases where stage was not known. *The figures in brackets refer to the occult cancers.

A total of 211 cases underwent needle localisation biopsy for impalpable (occult) lesions in the study population and in 80 of these cancer was detected. The pathology size distribution of these cancers is shown in Table I in bracketed figures. The screen detected cancers were predominantly pTIS or pT, the latter with a frequency of node positivity (15.4%) that was the lowest in the study.

The distribution of the cancers from the initial cohort and updates according to the histological classification is presented in relation to the population or subgroup source in Table II. This shows clearly the increased detection in the study group of noninvasive cancers (11.2%) and of invasive cancers with particular histological features (33.3%), an effect that is evidently attributable to screen detected cancers. Of particular note among screen detected cancers is the high proportion of invasive cancers with tubular and cribriform features, in either classical (8.7%) or variant (10.4%) forms.

The cancer histological characteristics are condensed into four categories in Table III to highlight the contrasting distribution of the cases in each category among the different population groups. In the screen detected cases, the proportion of invasive cancers with special histological features, either in classical or variant forms, was double that in controls. Of interest is the closely similar distribution of invasive cancers among non attenders to those in controls; conversely, those in the interval and other groups showed a high proportion of noninvasive and special type invasive cancers, as in the screen detected cases.

*Cancer characteristics as discriminants; size and lymph node status*

In view of the above findings it is relevant to investigate the ability of these same characteristics to discriminate between presentation categories, in particular the cancers of prevalence and incidence screens. The reasons for restricting populations in this analysis are given in Methods. Table IV shows the distribution of invasive cancers according to size (pT1, pT2) in the control and overall study population of the initial cohort only, giving also the breakdown into prevalence and incidence screens. Significant differences in the size distribution are evident between cancers of control and study populations ($P < 0.05$), and also between those of controls and cancers detected at both prevalence ($P < 0.05$) and incidence screens ($P < 0.01$). However, no significant size difference is noted comparing invasive cancers detected at prevalence and incidence screens.

**Table III** Histological categories of cancers according to population or presentation category (Initial cohort and updates)

| *Cateogry* | *Non-invasive* | *Special type* | *Variant special type* | *Not special type* | *Not known* | *Total* |
|---|---|---|---|---|---|---|
| Screen detected | 44 (15.2%) | 52 (19.1%) | 61 (21.2%) | 131 (45.4%) | 0 | 288 |
| Non attender | 3 (2.6%) | 14 (12.1%) | 13 (11.2%) | 66 (56.9%) | 20 (17.2%) | 116 |
| Interval + other | 8 (9.3%) | 13 (15.1%) | 10 (11.6%) | 48 (55.8%) | 7 (8.1%) | 86 |
| Control | 11 (3.2%) | 31 (9.1%) | 43 (12.7%) | 209 (61.7%) | 45 (13.3%) | 339 |

Three Phyllodes tumours were not included, one each in screen detected, interval and other and control populations. A further eight study population cases had unknown presentation.

**Table IV** Distribution of pT1 and pT2 invasive cancers according to presentation category (Initial cohort only)

| *Category* | *pT1* | *pT2* | *Total* |
|---|---|---|---|
| Prevalence screen | 51 | 21 | 72 |
| Incidence screen | 87 | 34 | 121 |
| Screen detected | 138 | 55 | 193 |
| Interval + other | 25 | 18 | 43 |
| Non attender | 21 | 19 | 40 |
| Study cases | 184 | 92 | 276 |
| Control cases | 85 | 65 | 150 |

**Table II** Histological type of cancer according to population or presentation category (Initial cohort and updates)

| *Histological type* | *Screen detected* | | *Study group* | | *Control group* | |
|---|---|---|---|---|---|---|
| Non invasive cancer: | 44 (15.2) | | 55 (11.2) | | 11 (3.2) | |
| Ductal | | 33 | | 44 | | 9 |
| Lobular | | 5 | | 5 | | 1 |
| Combined | | 4 | | 4 | | 1 |
| Unknown | | 2 | | 2 | | – |
| Invasive cancer | | | | | | |
| Special type: | 52 (18.1) | | 79 (16.2) | | 31 (9.1) | |
| Tubular/cribriform | | 25 | | 30 | | 8 |
| Mucoid | | 1 | | 4 | | 3 |
| Medullary | | – | | 1 | | 3 |
| Papillary | | 1 | | 1 | | – |
| Lobular | | 16 | | 24 | | 11 |
| Microinvasive | | 4 | | 4 | | 3 |
| Other | | 5 | | 15 | | 3 |
| Variants of special types: | 61 (21.2) | | 84 (17.1) | | 43 (12.7) | |
| Tubular/cribriform | | 30 | | 38 | | 22 |
| Lobular | | 12 | | 18 | | 9 |
| Medullary | | – | | 3 | | – |
| Others | | 18 | | 25 | | 12 |
| Unknown | | 1 | | | | |
| Not of special type: | 131 (45.5) | | 245 (50.0) | | 209 (61.7) | |
| Unknown | | – | 27 (5.5) | | 45 (13.3) | |
| Total | 288 (100) | | 490 (100) | | 339 (100) | |

Three Phyllodes tumours were not included, one each in screen detected, interval and other and control populations. A further eight study population cases had unknown presentation.

The same population groupings are maintained to compare node status in Table V. This shows separately the case numbers with node status known histologically to be either negative or positive as well as the total cases in the group. The salient features to note are that whilst significant differences occurred for node negativity between control and study populations (as well as subgroups), for node positivity only the prevalence screen differed significantly ($P < 0.05$) from controls. For both known node positivity and negativity no significant difference was found between cancers of prevalence and incidence screens. It appears therefore that whilst size and lymph node status are capable of distinguishing differences between study group and controls they give no useful measure of discrimination between cancers of prevalence and incidence screens.

### *Cancer characteristics as discriminants: histological characteristics*

The final comparisons (Table VI), again relating to the initial cohorts in both study and control populations, examine the distribution of the cancers in the three histological categories ST, VST and NST. In these assessments the significance tests allow for age at survey entry and apply only to the operable invasive cancers.

Comparison of study and control groups showed a significant difference ($P < 0.05$) in that more invasive cancers with special characteristics were present among the former. This difference was highly significant for the screen detected cancers ($P < 0.0001$). Examining further those cancers detected by screening, the prevalence cases differed highly significantly from controls ($P < 0.001$), while the incidence cases did not. Of particular note is the significant difference in histological type proportions between the prevalence and incidence screen cases ($P < 0.05$). In a logistic regression, the significance of histological type remained after allowance for size and node status, which themselves were not significant.

### *Comparisons of survival*

The ability of histological characteristics to distinguish subgroups of the study population suggest that these characteristics should be examined for influences on survival experience within the EBSP. Figure 2 shows the survival curves for control and screen detected cancers according to histological type in three categories. This shows clearly the survival advantage of cancers with special histological characteristics in both groups with almost 90% survivors at 7 years. However control group cancers lacking such features showed the poorest survival (60% at 7 years) and there was an identical experience for this category in non attenders to screening (data not shown). Among screen detected cases survival was also poorest for invasive cancers lacking special features (73% at 7 years), but survival in this group was significantly better than controls (logrank test, $P < 0.05$).

Using Cox's regression model the length of survival from diagnosis was significantly greater for the study cases relative to the controls (Hazard ratio = 0.54; 95% confidence interval = 0.39, 0.74), adjusting for age. This is not surprising, due to the lead time bias inherent in an analysis based on survival from date of diagnosis. However, when pathology size, node status and histological type are added the relative risk for the study population becomes non significant (HR = 0.98; 0.71, 1.36). The combination of tumour size (HR = 0.26; 0.16, 0.43) node negative status relative to positive (HR = 0.27; 0.16, 0.46) and histological type (HR = 0.46; 0.29, 0.73) explain the difference in survival between study and control cases. This suggests that a combination of these characteristics could provide an early measure of screening performance before the long term outcome in terms of mortality is known.

**Table V** Node status of pT1 and pT2 invasive cancers according to presentation category (Initial cohort only)

| *Category* | *Known negative* | *Known positive* | *Total* |
|---|---|---|---|
| Prevalence screen | 56 | 14 | 72 |
| Incidence screen | 85 | 31 | 121 |
| Screen detected | 141 | 45 | 193 |
| Interval + other | 25 | 15 | 43 |
| Non attender | 15 | 19 | 40 |
| Study cases | 181 | 79 | 276 |
| Control cases | 74 | 53 | 150 |

Totals include cases of unknown node status.

**Table VI** Histological category of operable invasive cancers according to presentation category (Initial cohort only)

| *Cateogry* | *Special type* | *Variant special type* | *Not special type* |
|---|---|---|---|
| Prevalence screen | 14 | 30 | 29 |
| Incidence screen | 21 | 26 | 72 |
| Screen detected | 35 | 56 | 101 |
| Interval + other | 8 | 5 | 34 |
| Non attender | 7 | 7 | 37 |
| Study cases | 50 | 68 | 172 |
| Control cases | 19 | 28 | 140 |

Note that the total number of cases differs from Tables IV and V as it refers to all operable invasive cancers.

### Discussion

Seven year mortality results have already been reported from this Trial (Roberts *et al.*, 1990) in which it was recognised that some bias was introduced into the study as a consequence of the 'cluster' effect of the randomisation process. Nevertheless through comparisons between the subgroups of the EBSP, this report gives particular attention to the utility of histological characteristics in the judgement of screening effect in advance of mortality influence.

Considering first the discriminant measure between prevalence and incidence screens, neither diminished cancer size nor node status was useful but histological features were. These findings have important implications for screening effect. The theory of screening predicts that prevalence screens are biased towards slow growing, less aggressive cancers (Cole & Morrison, 1980; Chamberlain, 1982). The Edinburgh results agree with this theory, as judged by the

**Figure 2** The survival curves from date of diagnosis of breast cancer for control (broken line) and screen detected (solid line) cases, according to histological type (abbreviations as in text).

high proportion of well differentiated special type invasive cancers and infrequent node positivity of the prevalence screen. Such features are less evident at incidence screens, however, and it is of some concern that a high node positive frequency was experienced in Edinburgh at the 'clinical only' incidence visits. One explanation would be that those cases were missed at the previous 'mammography and clinical' visits, which were themselves associated with low node positive frequency; another would be that the lesions were fast growing, aggressive cancers. To answer this would require blind retrospective radiological review. Yet concentration of this node positive effect among invasive cancers of 11 to 20 mm size range is of particular interest as it suggests a threshold effect for detection of some cancers that would need to be lowered to improve survival probabilities. Such observations have considerable importance in the context of the current 3-year interval between screens for the UK programme. The conclusions from the Nijmegen project favoured a 2-year interval (Peeters *et al.*, 1989) and a study of this aspect in the UK is one of the major new research initiatives (Chamberlain, 1990).

Nevertheless, histological node status offers some potential as an early index of screening benefit. In the Swedish Two Counties study, with a significant mortality benefit to the population offered screening, there was an unusually low lymph node positivity for invasive cancers less than 10 mm (Tabar *et al.*, 1987). This experience is remarkable for its divergence from other reported findings for the 1–10 mm invasive cancer size range, where node positivity seldom reduces below 20% (Frisell *et al.*, 1987; Carter *et al.*, 1989; Rosen *et al.*, 1989). Such a reduction was seen here only for the prevalence (and occult) screen cases but without significant overall mortality benefit (Roberts *et al.*, 1990). What may be relevant is to achieve a satisfactory yield of invasive cancers at incidence screens as well as to obtain reduction in the node positive proportion below that of the control population, particularly at 20 mm or less. For example, in the Östergötland component of the Two Counties trial the node positive proportion for all invasive cancers of incidence screens was 18% (controls 40%) with 85% of cases measuring 20 mm or less (controls 59%) (Hatschek *et al.*, 1989). The comparable figures for EBSP incidence screen invasive cancers were 28.5% and 47.9% respectively.

Histological discrimination between cancers at prevalence and incidence screens, noted here and previously (Anderson *et al.*, 1986) shows similarities with findings reported from the Östergötland project where histological grade of prevalence but not incidence screen cancers differed significantly from controls (Gröntoft, 1988). Preference for histological type characteristics has been emphasised here in view of previous associations with significant improved survival in symptomatic cases (Hutter, 1980; Dawson *et al.*, 1982; Dixon *et al.*, 1985), and for pragmatic reasons of biology and natural history. Such classifications have in the current prospective evaluation been translated successfully to survival differences in the EBSP at 7 years. These demonstrate the ready division on standard histological grounds into cancers with good (special type and variants) and poor (not special type) survival probabilities. The benefits of screen detection are apparent, particularly for invasive cancers with poor survival probabilities, but this could be attributed to an effect of lead time. Furthermore the possibility that variation in proportions of cancers in these histological categories at prevalence and incidence screens may reflect the natural history of cancer progression with time is a concept being explored. Certainly the adverse effect of non attendance, whether in terms of node positivity or histological characteristics, is readily perceived as a valid explanation for a dilutional effect in reducing the overall benefit to a population offered screening (Roberts *et al.*, 1990), stressing the importance of achieving high compliance. This analysis of pathological data from the EBSP suggests that early judgement of the screening effect ahead of mortality is possible. A combination of cancer size, node status and histological type is likely to give a more useful measure than size alone (Day *et al.*, 1989) in the early assessment of performance in breast cancer screening, with incidence screens being most informative.

Dr Maureen Roberts made fundamental and sustained contributions to this project but died on June 9th, 1989.

For their co-operation we thank the medical and ancillary staff at the Breast Screening Clinic, staff members of the Radiological and Surgical Units of Longmore Hospital, and colleagues of the Department of Pathology at the University of Edinburgh and Western General Hospital. We are grateful to Mr W. Lutz for his contribution in developing the computing and statistical aspects of this project. We also thank Miss Lesley Dunlop and Mrs Joyce Nicol for technical help; Mrs Carolyn Brown and Mrs Rosemary Williams for maintaining the pathology register and, with Mrs Joyce Garson, for providing secretarial support.

This work was financed by a grant from the Scottish Home and Health Department to the Edinburgh Breast Screening Project. (Committee members: Dr F. Alexander, Dr T.J. Anderson, Dr M.M. Andrew, Professor J. Best, Dr C. Brough, Mr U. Chetty, Dr W. Forbes, Professor A.P.M. Forrest (Chairman), Dr R. Gruer, Dr A. Huggins, Dr A.E. Kirkpatrick, Dr N.B. Louden, Mr W. Lutz, Dr U. Maclean, Dr M.M. Roberts, and Mr J.C. Duncan (project administrator)). T.J.A. was supported by grants from the Medical Research Council (G7803369, SPG 8214335). A grant was also provided by the Cancer Research Campaign (SP 1575, MMR); the pathology register computer was purchased with assistance from this grant.

### References

ANDERSON, T.J., LAMB, J., ALEXANDER, F.E. & 7 others (1986). Comparative pathology of prevalence and incidence cancers detected by breast screening. *Lancet* **i**, 519.

ANDERSON, T.J. (1989). Breast cancer screening. Principles and practicalities for histopathologists. In *Recent Advances in Histopathology*, Anthony, P., MacSween, R.N.M. (ed.) p. 43. Churchill Livingstone: Edinburgh.

AZZOPARDI, J.G. (1979). Problems in breast pathology. In *Major problems in Pathology*, Vol. 11, Bennington, J.L. (ed.) W.B. Saunders: London.

CARTER, C.L., ALLEN, C. & HENSON, D.E. (1989). Relation of tumor size, lymph node status, and survival in 24,740 breast cancer cases. *Cancer*, **63**, 181.

CHAMBERLAIN, J. (1982). Screening and natural history of breast cancer. In *Clinics in Oncology*, Vol. 1 (3), p. 679. Baum, M. (ed.) W.B. Saunders: London.

CHAMBERLAIN, J. (1990). (Chairman) Breast Screening Research Sub- Committee, United Kingdom Co-ordinating Committee on Cancer Research.

CHETTY, U., KIRKPATRICK, A.E., ANDERSON, T.J., LAMB, J., ROBERTS, M.M. & FORREST, A.P.M. (1983). Localisation and excision of occult breast lesions. *Br. J. Surg.*, **70**, 607.

COLE, P. & MORRISON, A.S. (1980). Basic issues in population screening for cancer. *J. Natl Cancer Inst.*, **65**, 1263.

COLLETTE, H.J.A., DAY, N.E., ROMBACK, J.J. & WARD, F. DE. (1984). Evaluation of screening for breast cancer in a non-randomised study (the DOM project) by means of a case-control study. *Lancet*, **i**, 1224.

DAWSON, P.J., FERGUSON, D.J. & KARRISON, T. (1982). The pathology findings of breast cancer in-patients surviving 25 years after radical mastectomy. *Cancer*, **50**, 2131.

DAY, N.E., WILLIAMS, D.R.R. & KHAW, K.T. (1989). Breast cancer screening programmes: the development of a monitoring and evaluation system. *Br. J. Cancer*, **59**, 954.

DIXON, J.M., PAGE, D.L., ANDERSON, T.J. & 5 others (1985). Long-term survivors after breast cancer. *Br. J. Surg.*, **72**, 445.

FISHER, E.R., GREGORIO, R.M., FISHER, B. & co-operating investigators (1975). The pathology of invasive breast cancer. *Cancer*, **36**, 1.

FRISELL, J., EKLUND, G., HELLSTRÖM, L. & SOMELL, A. (1987). Analysis of interval breast carcinomas in a randomized screening trial in Stockholm. *Breast Cancer Res. Treat.*, **9**, 219.

GRÖNTOFT, O. (1988). Staging and grading of invasive ductal carcinoma in a randomized population screened by mammography: the first and second screens. In *Screening for Breast Cancer*, p. 79. Day, N.E., Miller, A.B. (ed.) Hans Huber Publishers: Toronto.

HATSCHEK, T., FAGERBERG, G., STAL, O. & 4 others (1989). Cytometric characterization and clinical course of breast cancer diagnosed in a population-based screening program. *Cancer*, **64**, 1074.

HUTTER, R.V.P. (1980). The influence of pathologic features on breast cancer management. *Cancer*, **46**, 961.

PAGE, D.L., ANDERSON, T.J. (1987). *Diagnostic Histopathology of the Breast*. Churchill Livingstone: Edinburgh.

PALLI, D., ROSSELLI DEL TURCO, M., BUIATTI, E. & 4 others (1986). A case-control study of the efficacy of a non-randomised breast cancer program in Florence (Italy). *Int. J. Cancer*,. **38**, 501.

PEETERS, P.H.M., VERBEEK, A.L.M., HENDRIKS, J.H.C.L., HOLLAND, R., MRAVUNAC, M. & VOOIJS, G.P. (1989). The occurrence of interval cancers in the Nijmegen screening programme. *Br. J. Cancer*, **59**, 929.

ROBERTS, M.M., ALEXANDER, F.E., ANDERSON, T.J. & 7 others (1984). The Edinburgh randomised trial of screening for breast cancer: description of method. *Br. J. Cancer*, **50**, 1.

ROBERTS, M.M., ALEXANDER, F.E., ANDERSON, T.J. & 9 others (1990). Edinburgh trial of screening for breast cancer: mortality at seven years. *Lancet*, **335**, 241.

ROSEN, P.P., GROSHEN, S., SAIGO, E., KINNE, D.W. & HELLMAN, S. (1989). Pathological prognostic factors in stage I (TNM) and stage II (TNM) breast carcinoma: a study of 644 patients with median follow-up of 18 years. *J. Clin. Oncol.*, **7**, 1239.

SHAPIRO, S. (1977). Evidence on screening for breast cancer from a randomised trial. *Cancer*, **39**, 2772.

TABAR, L., DUFFY, S.W. & KRUSEMO, N.B. (1987). Detection method, tumour size and node metastasis in breast cancers diagnosed during a trial of breast cancer screening. *Eur. J. Can. Clin. Oncol.*, **23**, 959.

TABAR, L., GAD, A., HOLMBERG, L.H. & 9 others (1985). Reduction in mortality from breast cancer after mass screening with mammography. *Lancet*, **i**, 829.

UNION INTERNATIONAL CONTRA LE CANCRUM (1985). *TNM Classification of Malignant Tumours*. 4th Ed. UICC, Geneva.

VERBEEK, A.L.M., HENDRICKS, J.H.C.L., HOLLAND, R., MRAVUNAC, M., STUMMANS, F. & DAY, N.E. (1984). Reduction in breast cancer mortality through mass screening with modern mammography. (First results of the Nijmegen Project 1975–81). *Lancet*, **i**, 1222.